# Impact of a Local Vision Care Center on Glasses Ownership and Wearing Behavior in Northwestern Rural China: A Cluster-Randomized Controlled Trial

**DOI:** 10.3390/ijerph15122783

**Published:** 2018-12-08

**Authors:** Yue Ma, Yujuan Gao, Yue Wang, Haoyang Li, Lina Ma, Jiangchao Jing, Yaojiang Shi, Hongyu Guan, Nathan Congdon

**Affiliations:** 1 Center for Experimental Economics in Education (CEEE), Shaanxi Normal University, Xi’an 710119, China; mayue2871@126.com (Y.M.); gaoyujuanceee@163.com (Y.G.); wangyueceee@163.com (Y.W.); haoyangliceee@163.com (H.L.); shiyaojiang7@gmail.com (Y.S.); 2 Rural Education Action Program, Freeman Spogli Institute for International Studies, Stanford University, Palo Alto, CA 94305, USA; 3 School of Education, Shaanxi Normal University, Xi’an 710119, China; mln0426@foxmail.com; 4 School of Public Administration, Northwestern University, Xi’an 710127, China; jcjingchn@126.com; 5 State Key Laboratory of Ophthalmology and Division of Preventive Ophthalmology, Zhongshan Ophthalmic Center, Sun Yat-sen University, Guangzhou 510060, China; ncongdon1@gmail.com; 6 Centre for Public Health, School of Medicine, Dentistry, and Biomedical Sciences, Queen’s University Belfast, Belfast BT9 7BL, UK; 7 Orbis International, New York, NY 10018, USA

**Keywords:** visual impairment, vision center, cluster-randomized controlled, eyeglass ownership and wearing behavior

## Abstract

Visual impairment is common among rural Chinese children, but fewer than a quarter of children who need glasses actually own and use them. To study the effect of rural county hospital vision centers (VC) on self-reported glasses ownership and wearing behavior (primary outcome) among rural children in China, we conducted a cluster-randomized controlled trial at a VC in the government hospital of Qinan County, a nationally-designated poor county. All rural primary schools (*n* = 164) in the county were invited to participate. Schools were randomly assigned to either the treatment group to receive free vision care and eyeglasses, if needed, or control group, who received glasses only at the end of the study. Among 2806 eligible children with visiual impairment (visual acuity ≤ 6/12 in either eye), 93 (3.31%) were lost to follow-up, leaving 2713 students (45.0% boys). Among these, glasses ownership at the end of the school year was 68.6% among 1252 treatment group students (82 schools), and 26.4% (*p* < 0.01) among 1461 controls (82 schools). The rate of wearing glasses was 55.2% in the treatment group and 23.4% (*p* < 0.01) among the control group. In logistic regression models, treatment group membership was significantly associated with spectacle ownership (Odds Ratio [OR] = 11.9, *p* < 0.001) and wearing behavior (OR = 7.2, *p* < 0.001). County hospital-based vision centers appear effective in delivering childrens’ glasses in rural China.

## 1. Introduction

A large number of World Health Organization (WHO)-supported studies suggest that between 10 and 20 percent of school-aged children in developing countries suffer from refractive errors, with the highest rates occurring in China [1,2,3,4]. Nearly 50 percent of school-aged children worldwide who are visually impaired from refractive errors are in China [5]. Refractive errors can be easily detected with visual acuity (VA) screening and safely corrected [5] with a pair of prescription eyeglasses [1]. Although this simple intervention is very easy to implement, in developing settings such as rural China, the rates of eyeglasses ownership or wearing eyeglasses are less than 25 percent among school-age children with poor vision [6,7].

There are a number of reasons for the low rates of eyeglasses ownership and wear in low-resource settings. One barrier is cost, although this is primarily an issue only for the lowest income families in rural China [8]. In reality, a large number of families are able to spend money on vision care for their children in low income areas [9]. Another important reason for the low rate of eyeglass ownership and wear among school-aged children may be a lack of high-quality vision care services [10]. Study findings suggest that about 625 million people globally suffer from vision problems because they lack access to vision care facilities, equipment and skilled vision care practitioners [11,12,13].

As an indication of the importance of vision care, the Chinese government announced on 30 August 2018 plans for a comprehensive national children’s myopia management project, supported in an editorial by national leader Xi Jinping. Actions will be coordinated among eight central government bodies under the leadership of the Education Ministry [14]. For China’s central government to effectively implement such a project, it is necessary to explore ways to provide low-income communities with high-quality vision care services and eyeglasses

The community-based vision center (VC) is a popular model for non-governmental organizations (NGOs) and local governments to provide low-income communities with high-quality vision care services and eyeglasses [11,14,15,16]. Offering eye examinations, refraction, and optical dispensing, community based VCs are a sustainable way overcome both supply and demand-side the barriers that prevent local communities and target individuals with uncorrected refractive error from accessing affordable eye care services and eyeglasses [11,14,15,16,17].

Despite the popularity of VCs, there is only one study has evaluated the quality and effect of the vision care services they deliver in rural China [18]. The study showed that a VC had a positive impact on children’s eyeglass ownership and wearing behavior, but because the location was in a small, relatively affluent rural county, we do not know whether this model would be effective in other settings.

In this study, we conducted a cluster-randomized control trial (RCT) to evaluate the effect of a county hospital-based VC on eyeglass ownership and wearing behavior among rural school-aged children. We hypothesized that access to the VC-provided optometric services would lead to an increase in eyeglasses ownership, and consequently higher rates of wearing eyeglasses (primary outcome). Both to reduce costs and to promote eyeglass wearing, we involved the teachers of sample students in the VA screening program, as randomized trials conducted elsewhere have shown that the involvement of teachers in school-based programs can significantly increase rates of eyeglass ownership and eyeglass wearing [7,18].

## 2. Materials and Methods

The protocol for this study was approved in full by the Institutional Review Boards (IRB) (Protocol ID 24847) at Stanford University (Palo Alto, CA, USA) and the Zhongshan Ophthalmic Center (Guangzhou, China). Permission was received from local Boards of Education in each region, and the principals of all schools. All participating children gave oral assent prior to baseline data collection, and legal guardians gave written consent for their children’s involvement in the study. The principles of the Declaration of Helsinki were followed throughout.

### 2.1. Vision Center Set-up and Staff Training

We established a VC in the county level hospital of Qinan, a nationally-designated poor county in rural northwestern China. The total population of Qinan County is 591,245 [19] and the per-capita Gross Domestic Product (GDP) of the county is US$1573. Qinan ranks 74th out of 87 counties in Gansu Province [20], the poorest of China’s 31 administrative regions [21]. Prior to the establishment of the VC, the county had three private vision care providers (all located in the county seat) and no public vision care services.

The VC was established in collaboration with the provincial and prefectural Bureaus of Education. The goal of this project was for Qinan to act as a model county for other poor counties in rural China, with eventual upscaling to provide high-quality vision care services to all rural school-aged children in China.

The Qinan County Hospital chose three employees (one ophthalmologist and two ophthalmic nurses) to staff the VC. These individuals underwent one month of refraction training (September–October 2014) at one of China’s leading institutions, Zhongshan Ophthalmic Center in Guangzhou. At the end of the training program, all three trainees were certified as qualified refractionists by China’s Ministry of Labor and Social Security. After this didactic training, the three returned to their home county to perform vision screenings and refractions for hundreds of children from local schools as a part of a one-month practical training program. During this time, they also received instruction in eyeglass dispensing. A consultant from the Zhongshan Ophthalmic Center in Guangzhou provided professional training on topics including inventory control and record-keeping.

### 2.2. Sampling and Eligibility Criteria 

All rural primary schools in Qinan County werecluded in in this study. Within each school, we examined all children in Grades 4–6. All children with uncorrected VA of ≤6/12 in either eye without wearing eyeglasses were enrolled in the trail.

### 2.3. Treatment and Experiment Design

The project was implemented as a cluster-RCT, with randomization at the township level. Schools were randomly assigned to the control or treatment group by township (cluster size 8–9). In the treatment group, all children who failed the VA screening were referred to the VC for refraction and free eyeglasses as needed from the beginning of the school year (Fall, 2014) to the end of school year (Spring, 2015). In the control group, children did not receive the screening intervention until after the evaluation at the end of the study. Members of the study team from Shaanxi Normal University, in Xi’an, China, conducted randomization at the beginning of project.

Power calculations were conducted using Optimal Design software [22] for cluster randomization and binary outcome (wearing glasses vs not wearing glasses). Based on our earlier experiments with primary school-aged children, we assumed an estimated rate of wearing glasses at approximately 20% in the Control and 55% in the Treatment group, and a 30% prevalence of refractive error. We determined that 17 towns with 532 students per town (with 165 expected to have refractive error) would provide 90% power to detect the expected difference between groups with an alpha error of 0.05, and intra-class correlation of 0.15.

Teachers participated in a one-day training on VA screening by VC staff. Teachers in both the control and treatment groups received this training before schools participated our program. All children’s visual acuity was measured only once before schools participate our program. We did not collect children’s visual acuity as a follow up outcome. In the treatment group, teachers screened the children during the intervention (before the endline survey). In the control group, teachers received professional training on visual acuity screening only after endline. Based on the results of the teacher screening, parents of children with uncorrected VA of ≤6/12 in either eye received a letter that provided a description of the program and an invitation to bring their children to the VC for free vision care services. These services included free rescreening, refraction and, if needed, a free pair of prescription eyeglasses. Additionally teachers who conducted the initial screening were asked to send a list of students who failed the screening to the VC staff. VC staff then made follow-up phone calls to both the homeroom teachers and the families of these students to encourage parents to bring their children to the VC for services. All children’s visual acuity was measured only once, by their teachers; there was no follow-up visual acuity screening, and no members of the research team conducted any visual acuity screenings. In our analyses, when we compare baseline data between the intervention and control groups, we are comparing students who have already received VA screening with students who have not.

All participants (students, parents, and teachers) as well as VC staff were not informed of the study design or group assignment. Participants were told only that this was a study of vision care among rural primary school children and were masked to group assignment at the time of the follow up outcome assessment. The control group followed the same referral pathway as the treatment group but only began after the endline survey was completed. Because teachers did not know how to conduct VA screenings until they received the one-day professional training by VC staff, and because control group teachers received professional training on VA screening only after endline, contamination across treatment arms was nearly impossible.

### 2.4. Data Collection

In September 2014, teachers conducted a survey of socioeconomic data among 4–6th grades children in both treatment and control groups. The socioeconomic survey collected data on gender, eyeglass ownership, distance between town and county seat, and parental migration and educational attainment (all factors likely to influence uptake of spectacles [23]).

The follow-up survey was conducted in the June 2015. Our primary outcome was self-reported glasses wearing behavior at the time of the follow-up survey. Students could report wearing glasses “always,” “only for studying,” or “usually not worn.” Children were also asked to state whether they owned eyeglasses or not when they were screened by teachers.

### 2.5. School-Based Visual Acuity Assessment 

Visual acuity was tested separately for each eye without refraction at 4 meters, using Early Treatment Diabetic Retinopathy Study (ETDRS) charts (Precision Vision, La Salle, IL, USA). All VA screenings were conducted in a well-lighted, indoor area [24]. Children who owned eyeglasses were requested to bring them to school, and during the screening their visual acuity was tested both with and without eyeglasses. We defined visual acuity for one eye as the lowest line on which 4 of 5 optotypes could be read correctly. If a student could not correctly read the top line at 4 m, was tested at 1 m, and the measured visual acuity was divided by 4. We used Snellen equivalents (Early Treatment Diabetic Retinopathy Study charts) when teachers screened at school. In order to analyze it in this paper, we converted students’ VA to logMAR.

### 2.6. VC-Based Examination and Refraction

All eye refractive error services were provided by one of the three trained refractionists in the established VC, following China’s “National Guidelines for Vision Care” for prescribing eyeglasses. First, a refractionist discussed the child’s history of refractive error services with the parent, after which the refractionist performed another VA screening as described above. Based on the results of this re-screening, children with an uncorrected VA of ≤6/12 in either eye underwent cycloplegia with up to three drops of cyclopentolate 1%, preceded by a drop of proparacaine hydrochloride 0.5% to prevent accommodation and inaccurate refraction. All center-based VA testing, including cycloplegia, was conducted in a single visit for each child. Children then underwent automated refraction (Topcon KR 8900, Tokyo, Japan) with subjective refinement by the refractionist. Children whose refractive error results met cutoffs shown to be associated with significantly greater improvement in visual acuity when corrected (Myopia <= −0.75 diopters [D], Hyperopia >= +2.00 D or Astigmatism [Non-spherical refractive error] >= 1.00 D) and whose VA could be improved to >6/12 in both eyes with refraction [25] received a pair of free eyeglasses.

Finally, before making eyeglasses for the child, the refractionist measured the child’s interpupillary distance. If the child owned a pair of glasses, the lens power of the child’s original eyeglasses were also measured at this time. The VC had roughly 10 different styles of child-friendly frames, and children were permitted to choose whichever frames they liked the most.

### 2.7. Statistical Analysis

We used Stata 14.2 (Stata Corp., College Station, TX, USA) to perform all of our analyses, including the calculation of robust standard errors to adjust for clustering by township. Baseline eyeglasses ownership was defined as whether the children have a pair of prescription eyeglasses at school, after being asked to bring them. Refractive power was defined as the spherical equivalent: spherical power plus half the cylindrical power.

For intention-to-treat (ITT) analyses, with eyeglasses ownership and wearing behavior as outcomes, we used a logistic regression model to estimate the odds ratio for the treatment group, adjusting for baseline glasses ownership and other covariates. First, we used a one-way variance regression model to estimate the intra-class correlation coefficient as a measure of clustering of eyeglass ownership and wearing behavior within each township. To evaluate our hypothesis, we used the outcomes of eyeglasses ownership and self-reported eyeglass wearing behavior (comparing “only for studying” or “always,” to “mostly not worn”). Second, we used a multiple logistic regression model to measure the VC treatment effect on self-reported eyeglasses wearing behavior and ownership at the endline survey, adjusting for other baseline student characteristics. These characteristics included variables associated with wear at the end of the study at *p* < 0.20 (baseline eyeglass ownership, baseline uncorrected VA, baseline math score, parental education, and parental migration) as well as those that we felt were important on a theoretical basis (sex, boarding status, distance between town and county seat).

In order to improve the efficiency of estimation, we used a logistic regression to impute the following missing data points for baseline variables (using Stata14.2, as described by Royston): both parents out-migrated for work (*n* = 43) and at least one parent has 9 years education (*n* = 64) [26]. The independent variables used to conduct the imputation included all variables that were not missing. For each variable, different models were used to select the independent variables based on their predictive value and availability of data. The multiple imputation approach created 20 copies of the data in which missing values were imputed by chained equations. Final results were obtained by averaging these 20 data sets using Rubin’s rules, which ensured that the standard errors for all regression coefficients take into account the uncertainty in the imputations as well as uncertainty in the estimation.

## 3. Results

**Figure 1:**
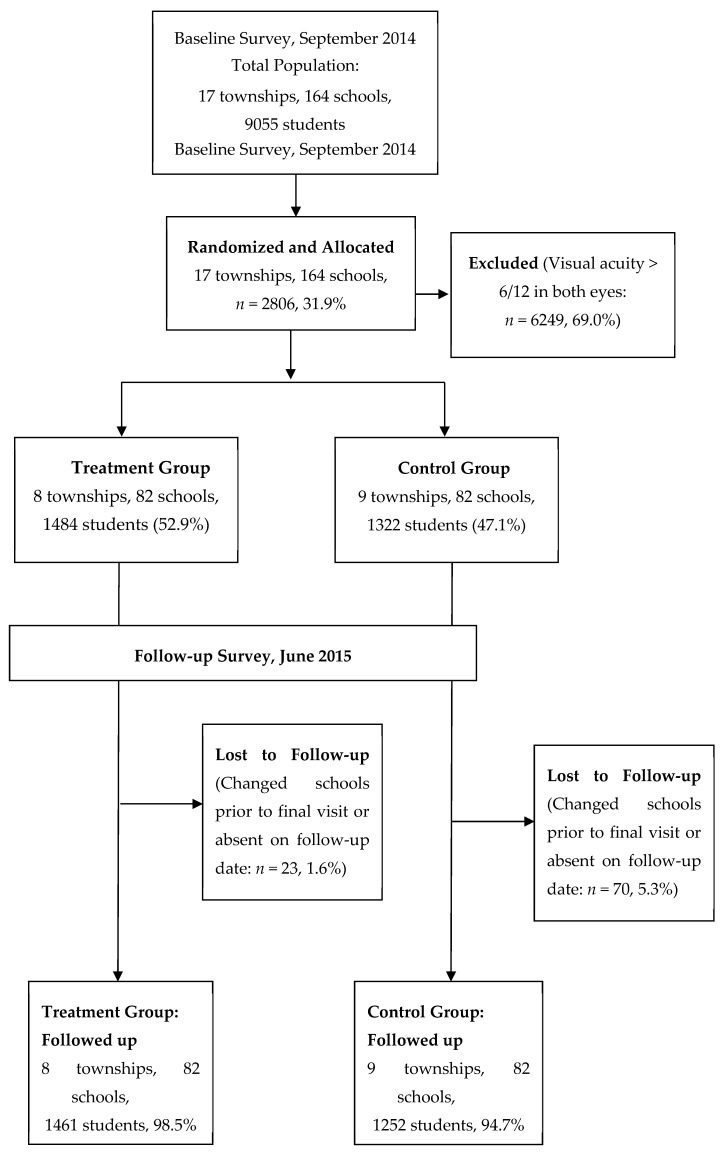
Flow Enrollment Chat and Progress of the Trial.

Of the 9055 sampled children screened at 164 selected schools in 17 townships, 6249 (69.0%) passed school-based vision screening, and 2806 (31.0%) failed, indicating an uncorrected visual acuity of ≤6/12 in either eye, making them eligible for the study. The median visual acuity at baseline for eligible students was 6/24 in the better eye. All eligible children were of Han Chinese ethnicity, 1493 (53.2%) were girls. A total of 82 schools (8 townships, 1484 children (52.9%)) were randomly assigned to the treatment group and 82 schools (9 townships, 1322 children (47.1%)) to the control group (Figure 1).

Among 2806 children enrolled and assigned to study groups, 93 (3.31%) were lost to follow-up due to a change of schools prior to the final visit or being absent on the follow-up date. This left a final analytic sample of 2713 students: 1461 students (98.5%) in the treatment group and 1252 students (94.7%) in the control group (Figure 1). There was no significant difference in individual-level cluster-level variables between children in the treatment and control groups or between children with and without follow-up (Table 1).

Our unadjusted results (Table 2) show that rates of both owning eyeglasses and wearing eyeglasses at the final visit were higher in the treatment group compared to the control group (ownership: 68.6% vs. 26.4%, difference = 42.2%, *p* < 0.01; wearing behavior: 55.2% vs. 23.4%, difference = 31.8%, *p* < 0.01).

Predictors of eyeglass ownership at the final visit in full multivariate models included membership in the treatment group (OR = 11.9, *p* < 0.01), baseline ownership (OR = 31.8, 95% CI = 10.2–98.9, *p* < 0.001), baseline mathematics score (OR = 1.19, 95% CI = 1.08–1.31, *p* < 0.001), and uncorrected VA (children with worse VA were more likely to own eyeglasses: OR = 31.8, 95% CI = 10.2–98.9, *p* < 0.001). The results were very similar for eyeglass wearing behavior at the final visit (main outcome), with membership in the treatment group, baseline eyeglass ownership, baseline mathematics score, and worse uncorrected VA as the only variables associates with glasses wearing behavior (Table 3).

## 4. Discussion

### 4.1. Principal Findings

Using the ITT estimator, we found a statistically significant improvement in eyeglasses ownership and wearing behavior among 4–6th grade rural children randomly assigned to the treatment group (versus the control group) and referred to a VC after preliminary school-based vision screening, where they received free refraction and eyeglasses, if needed. Among all students, only one sixth (16.6%) owned eyeglasses at baseline. The ownership of eyeglasses among the control group also increased from 17.01% in baseline to 26.4% by the end of study. That’s because the average severity of impairment (using LogMAR as a measure) rises steadily overtime among school-age children [27].

While the rate of ownership of glasses does increase from baseline to endline, it does not keep up with the rate of increase in visual impairment. Overall, the attrition rate is low in our trial. In the attrition test, only one control variable (standardized math score) out of seven variables was significantly different (*p* = 0.039) between the analytic sample and the attrited sample. This is considered acceptable in an RCT set.

The significantly-improved rates of owning and wearing eyeglasses were maintained over the course of the full school year. The positive results of this RCT have important implications for future vision care programs for school-aged children in rural areas. Our findings suggest that setting up a vision care model (a school-based vision screening, hospital-based VC) can significantly increase the uptake of vision care services in China’s poor rural counties. Because we considerate that the intervention in our VC model includes school-based teacher screening, free rescreening, refraction and, if needed, a free pair of prescription eyeglasses. We are unable to separate the distinct impacts of each component intervention on its own.

### 4.2. Comparison with Other Studies

Our research team previously established a hospital-based VC in Yongshou County, a nationally-designated poor county in rural Shaanxi Province, also in northwestern China. A study of this VC, also conducted by our research team, found a significant effect of rural hospital-based VCs on self-reported glasses ownership and glasses wearing [18]. Qinan is a larger county than Yongshou: its population (591,254) [19] is nearly three times that of Yongshou (208,065) [28]. Qinan is also a poorer county than Yongshou: the per-capita GDP of Qinan (US$1573) [19,20] is barely a third of that in Yongshou (US$4239) [28,29]. Additionally, the rate of baseline glasses ownership among children in Qinan (17%) is just over half that of Yongshou (29%). It is very important for program planners to have high-quality evidence of the effectiveness of the VC model in China’s largest and poorest rural counties.

Previous vision care studies that provided free eyeglasses [6,10,30] have often found low rates of eyeglasses ownership and eyeglasses wearing, even when educational interventions to promote eyeglasses use are provided in addition to free eyeglasses [6,31]. Studies on this subject have assessed the use of eyeglasses over periods between one month to one year, and these studies have relied on several different measures, including self-reported eyeglasses wearing behvaior [31,32,33] estimates by parents, teachers, or health professionals [34] and directly observed wearing behavior [6,10,30,35,36,37]. Observed rates of eyeglasses wearing are often lower than other measures, ranging from 13% to 41% [8,10,29,30,36,37]. Although a few studies report higher rates of eyeglasses wearing (e.g., 46% by Keay et al. [37] in China, 56% by Vincent et al. [31] in Thai refugee camps, and 58% by Von-Bischhoffshausen et al. in Chile [31], and 69% in China by Yi et al. [7]), all have low (58–76%) rates of follow-up or assessment times as short as 1 month after eyeglasses distribution [31,33,37], or involved additional interventions such as teacher incentives [7].

Additionally, past studies have found that including teachers as a part of school-based vision screening programs can have a positive effect on glasses ownership and wear among school-aged children [7,18]. This study incorporated teachers as vision screeners, which may have increased the treatment effects of this study. However, because teacher participation was a part of our treatment arm and there was no comparison arm that did not use teachers for vision screening, it is not possible to identify the sole effect of teacher participation on student attendance or glasses wear.

### 4.3. Strengths and Limitations of the Study

The strengths of this study include its large RCT design and its successful collaboration with local Bureaus of Education and county hospital to implement the study. Additionally, the choice to establish the VC as part of a county hospital makes this study relevant to future vision care policies and government initiatives. All of these increase confidence in the findings and their relevance to actual programs.

Weaknesses, however, must also be acknowledged. First, all of our sample schools were enrolled from one county in rural northwestern China. This limits the external validity of the study. Future studies are needed to examine this model in poor counties in other regions of China. Second, children with and without follow-up differed in academic performance (measured by baseline mathematics—Table 1). However, follow-up rates saw no significant differences between the treatment and control group, and we controlled for mathematics score in our regression models. In addition, our outcome variables (glasses ownership and wearing rates) relied on self-report data, which may overestimate actual behavior [6,7]. Also, delaying the referral to the VC essentially delayed visual correction for myopic students in the control group. While we are unable to control for these (possible) unobserved trends, assuming that they do hold in our data, it would mean that our estimates of impact of the VC are underestimates, and that the real impact may actually be larger than what we observe in this study. Finally, we did not attempt to calculate the program cost effectiveness, which may be valuable to future research and policy design.

## 5. Conclusions

Despite the limitations, this study makes a valuable contribution by testing the county hospital-based model of refractive service delivery in a large and very poor rural county. Our findings suggest that this model may be effective for providing vision care to school-age children in rural China and other countries with a high prevalence of refractive error.

## Figures and Tables

**Table 1:** Baseline characteristics of children with correctable refractive error, by group assignment.

Variable	Analytic Sample (*n* = 2713)			Full Sample (*n* = 2806)			Difference Between Children with and without Follow-up		
	Treatment Group (*n* = 1461)	Control Group (*n* = 1252)	*p*-Value	Treatment Group (*n* = 1484)	Control Group (*n* = 1322)	*p*-Value	Completed Follow-up (*n* = 2713)	Without Follow-up (*n* = 93)	*p*-Value
Male, *n* (%)	660 (45.17)	560 (44.73)	0.860	669 (45.08)	594 (44.93)	0.952	1221 (44.97)	43 (46.24)	0.770
Baseline standardized math score, mean (SD)	0.25 (1.03)	0.01 (1.00)	0.083	0.24 (1.03)	0.00 (1.00)	0.066	0.14 (1.02)	−0.11 (1.00)	0.039
Distance between town and county seat, mean (SD), km	30.95 (15.40)	24.37 (26.59)	0.641	30.90 (15.40)	24.37 (26.62)	0.645	27.91 (21.56)	25.14 (24.77)	0.652
Visual acuity of better eye, mean (SD)	0.44 (0.27)	0.46 (0.28)	0.186	0.43 (0.27)	0.46 (0.28)	0.142	0.45 (0.27)	0.44 (0.29)	0.852
Owned eyeglasses at baseline, *n* (%)	238 (16.29)	213 (17.01)	0.789	243 (16.37)	227 (17.17)	0.784	450 (16.62)	19 (20.43)	0.678
One or both parents with ≥9 years of education, *n* (%)	302 (20.67)	240 (19.25)	0.629	309 (20.75)	256 (19.36)	0.624	543 (20.01)	21 (22.58)	0.526
Both parents out-migrated for work, *n* (%)	221 (15.06)	177 (14.14)	0.835	227 (15.30)	181 (13.69)	0.719	396 (14.63)	11 (11.83)	0.561

Note: Analytic Sample: The sample used in analysis and all attended follow-up survey; Full Sample: all sample who has attend baseline survey and not necessarily involved in the follow-up survey.

**Table 2:** Self-reported Eyeglass Ownership and Wearing behavior by Study Group at Final Visit among 2713 Children.

Outcome Variables at Final Visit	Control Group (*n* = 1461)	Treatment Group (*n* = 1252)	*p*-Value ^a^
Self-reported eyeglass ownership, *n* (%)	386 (26.4%)	859 (68.6%)	<0.01
Self-reported eyeglass wearing behavior, *n* (%)	342 (23.4%)	691 (55.2%)	<0.01

Note: ^a^ Paired 2-sample *t*-test.

**Table 3:** Logistic Regression Model of Potential Factors Associated with Self-reported Ownership and Wearing behavior (Main outcome) of Glasses at End of Study.

Variable	Self-Reported Eyeglass Ownership at End of Study, (*n* = 2713)				Self-Reported Eyeglass Wearing Behavior at End of Study, (*n* = 2713)			
	Univariate Adjusted for Baseline Ownership ^d,e^		Full Model ^d,e^		Univariate Adjusted for Baseline Ownership ^d,e^		Full Model ^d,e^	
	Odds Ratio	*p*-Value	Odds Ratio	*p*-Value	Odds Ratio	*p*-Value	Odds Ratio	*p*-Value
	(95% CI) ^a,c^		(95% CI)		(95% CI)		(95% CI)	
Treatment group	10.61	<0.001	11.89	<0.001	6.28	<0.001	7.21	<0.001
	(6.22–18.12) ^b^		(6.77–20.89) ^b^		(3.35–11.76) ^b^		(3.74–13.88) ^b^	
Male sex, no. (%)	1.02	0.838	-		1.04	0.527	-	
	(0.86–1.20)		-		(0.91–1.19)		-	
Baseline standardized mathematics score	1.32	0.001	1.19	<0.001	1.28	<0.001	1.15	0.005
	(1.12–1.56) ^b^		(1.08–1.31) ^b^		(1.10–1.49) ^b^		(1.04–1.27) ^b^	
Distance between town and county seat	1.00	0.872	-		1.00	0.900	-	
	(0.97–1.03)		-		(0.98–1.03)		-	
Owned eyeglasses at baseline	23.54	<0.001	31.82	<0.001	14.88	<0.001	14.47	<0.001
	(9.06–61.18) ^b^		(10.24–98.91) ^b^		(6.84–32.39) ^b^		(4.81–43.56) ^b^	
Visual acuity of better eye	3.51	<0.001	6.74	<0.001	6.19	<0.001	10.36	<0.001
	(2.20–5.60) ^b^		(3.60–12.62) ^b^		(3.42–11.20) ^b^		(5.34–20.08) ^b^	
One or both parents with ≥9 years of education	1.144	0.332	-		1.27	0.113	1.18	0.281
	(0.87–1.50)		-		(1.00–1.70)		(0.87–1.61)	
Both parents out-migrated for work	1.05	0.788	-		0.87	0.428	-	
	(0.73–1.53)		-		(0.62–1.22)		-	

Note: ^a^ CI = 95% confidence interval, reported in parentheses; ^b^ comparisons for which the 95% CI for effect size does not cross 1; ^c^ except for the regression coefficient for the rate of ownership of eyeglasses at baseline (simple logistic regression), coefficients for the different variables are for multiple models with glasses wearing behavior/ownership as the dependent variable, adjusted for baseline eyeglasses ownership; ^d^ robust standard errors are adjusted for clustering at the town level; ^e^ all estimates include fixed effects for grade.
